# Memory Boost for Recurring Emotional Events Is Driven by Initial Amygdala Response Promoting Stable Neocortical Patterns across Repetitions

**DOI:** 10.1523/JNEUROSCI.2406-23.2025

**Published:** 2025-02-13

**Authors:** Valentina Krenz, Arjen Alink, Benno Roozendaal, Tobias Sommer, Lars Schwabe

**Affiliations:** ^1^Department of Cognitive Psychology, Institute of Psychology, University of Hamburg, Hamburg 20146, Germany; ^2^Department of Psychology and Neuroscience, Boston College, Chestnut Hill, Massachusetts 02467; ^3^Institute of Systems Neuroscience, University Medical Center Hamburg-Eppendorf, Hamburg 20251, Germany; ^4^Department of Cognitive Neuroscience, Radboud university medical center, Nijmegen, 6500 HB, The Netherlands; ^5^Donders Institute for Brain, Cognition and Behaviour, Radboud University, Nijmegen 6525 EN, The Netherlands

**Keywords:** amygdala, emotional memory, hippocampus

## Abstract

Emotionally arousing events are typically vividly remembered, which is generally adaptive but may contribute to mental disorders such as post-traumatic stress disorder. Previous research on emotional memory focused primarily on events that were experienced only once, leaving the memory mechanisms underlying repeatedly encountered emotional events largely unexplored. Here, we aimed to elucidate the brain mechanisms associated with memory for recurring emotional events. Specifically, we sought to determine whether the memory enhancement for recurring emotional events is linked to more variable neural representations, as predicted by the encoding-variability hypothesis, or to more stable representations across repetitions, as suggested by a memory reinstatement account. To investigate this, we repeatedly presented healthy men and women with images of emotionally negative or neutral scenes during three consecutive runs in an MRI scanner. Subsequent free recall was, as expected, enhanced for emotional compared with neutral images. Neural data showed that this emotional enhancement of memory was linked to (1) activation of the amygdala and anterior hippocampus during the initial encounter of the emotional event and (2) increased neural pattern similarity in frontoparietal cortices across event repetitions. Most importantly, a multilevel-moderated mediation analysis revealed that the impact of neocortical pattern stability across repetitions on emotional memory enhancement was moderated by amygdala activity during the initial exposure to the emotional event. Together, our findings show that the amygdala response during the initial encounter of an emotional event boosts subsequent remembering through a more precise reinstatement of the event representation during subsequent encounters of the same event.

## Significance Statement

Despite extensive research on emotional memory, the mechanisms underlying memory formation for recurrent emotional events remain elusive. We show that amygdala and anterior hippocampal activity is most prominent during the initial exposure to an aversive stimulus and decreases markedly with repeated exposure. Neocortical representation patterns of subsequently recalled emotional events, however, are more stable across the repeated encounters of emotional (vs neutral) events, in line with a memory reinstatement account. Notably, this increased neocortical pattern stability was driven by the amygdala response during the initial exposure to an emotional event. These findings provide novel insights into the mechanisms involved in memory formation for recurrent emotional events, with potential implications for complex post-traumatic stress disorder characterized by multiple traumatic exposures.

## Introduction

Emotionally arousing events are often well remembered (McGaugh, 2006), aiding the avoidance of future threats (Baczkowski et al., 2023). However, their persistent, vivid recollection may affect mental well-being and contribute to psychopathology (Brewin et al., 2010). Importantly, emotional events may be encountered repeatedly, and memories are shaped through repeated (re)encoding processes (Nadel and Moscovitch, 1997; Dudai, 2012). For instance, trauma survivors frequently re-encounter trauma-related stimuli reinstating the original memory (Ehlers and Clark, 2000) and are at an increased risk of re-experiencing similar traumatic incidents (Kessler et al., 2017). While decades of research provided valuable insights into the memory formation for emotional events experienced just once (Murty et al., 2011; Dahlgren et al., 2020), the evolution of memory across repeated encounters to the same emotional event remains elusive.

Repeated study is well known to strengthen memory (Ebbinghaus, 1885). One prominent theory of the enhanced memory after repetition, referred to as encoding-variability theory (Estes, 1955; Johnston, 1976), postulates that each time we encounter an event, this event is encoded differently due to variations in the temporal or spatial context. These variations would multiply the access routes to the memory and hence promote its future recall (Johnston, 1976). Alternatively, each encounter may reactivate and thus strengthen the memory representation formed during previous encoding (Thios and D’Agostino, 1976). Extant studies indicate that memory formation is successful when the same neocortical representations are reactivated across subsequent encounters rather than when patterns of activation are more variable across repetitions (Xue et al., 2010, 2013; Feng et al., 2019). Importantly, these studies included only neutral events and, thus, whether the evolution of emotional memory across repeated exposures is based on more stable or more variable activation patterns is completely unknown.

Emotional compared with neutral events recruit specific neural mechanisms that enable their preferential storage in memory. A key role in this process is attributed to noradrenergic arousal-induced activation of the amygdala, which modulates memory processes in the hippocampus and neocortex (McGaugh, 2000; Fastenrath et al., 2014). This arousal-related mechanism specifically promotes the consolidation of emotional long-term memories (Cahill and McGaugh, 1998; McReynolds and McIntyre, 2012). However, enhanced recall of emotional over neutral events is also evident immediately after initial encoding, before a consolidation delay (Talmi et al., 2007; Murty et al., 2011; Talmi and McGarry, 2012; Schümann et al., 2018). This immediate emotional memory enhancement has been attributed to preferential processing and increased attention at encoding, promoting the immediate free recall of these items (Talmi, 2013). Furthermore, the anterior hippocampus, strongly connected to the amygdala (Pitkänen et al., 2000; Richardson et al., 2004; Tillman et al., 2018), has been repeatedly linked to successful encoding of emotional memory (Murty et al., 2011). Importantly, both the amygdala (Ranganath and Rainer, 2003) and anterior hippocampus (Strange et al., 1999; Cowan et al., 2021) have been implicated in novelty detection, which may point to a role of the anterior mediotemporal lobe (MTL) specifically when emotional events are experienced for the first time.

Here, we aimed to elucidate the neural mechanisms underlying memory enhancement for repeatedly encountered emotional events. Specifically, we investigated whether the emotional enhancement of memory is due to more stable or variable neural patterns and whether these dynamics over repeated encounters are driven by initial anterior MTL responses. To this end, participants viewed images of emotionally negative or neutral scenes in three consecutive runs in an MRI scanner, followed by an immediate free recall test. We expected an emotional memory enhancement associated with enhanced anterior MTL activity at initial encounter and more consistent pattern representations across repeated encounters in visual and frontoparietal cortices. Using moderated mediation analyses, we further examined the link between transient anterior MTL involvement and stable neocortical activation patterns for recurring emotional events that were subsequently remembered.

## Materials and Methods

### Participants and design

One hundred and nine healthy volunteers (55 males, 54 females; age: M = 24.09 years, SD = 3.92 years) participated in this experiment. Exclusion criteria were checked in a standardized interview and comprised a history of any psychiatric or neurological diseases, medication intake or drug abuse, as well as any contraindications for MRI measurements. All participants provided informed consent before taking part in the experiment and received monetary compensation for participation. The study protocol was approved by the ethics committee of the Medical Chamber Hamburg (PV5480) and was in accordance with the Declaration of Helsinki. Six participants had to be excluded from the analysis because of technical failure (*n* = 1), missing data for at least one of the experimental tasks (*n* = 2), or falling asleep during MRI scanning (*n* = 3), thus resulting in a final sample of 103 right-handed young adults (51 males and 52 females; age: M = 24.08 years, SEM = 0.39 years). A sensitivity analysis using MorePower 6.0 (Campbell and Thompson, 2012) confirmed that this sample size is sufficient to detect a medium-sized effect (ƞ^2^ = 0.07) for a 2 (memory) × 2 (emotion) × 3 (run) interaction with a power of 0.95 (*α* = 0.05).

### Experimental procedure

This study is part of a larger project investigating modulators of time-dependent systems consolidation and memory transformation processes (Krenz et al., 2021, 2023). Therefore, shortly before stimulus presentation in the MRI, participants received orally either placebo or 20 mg yohimbine (double-blind), an α_2_-adrenoceptor antagonist leading to a temporally delayed increase in noradrenergic stimulation. The timing of the drug administration was chosen based on previous studies (Schwabe et al., 2012; Kluen et al., 2017) and the known pharmacodynamics of yohimbine, showing that a significant drug action can be expected ∼60 min after drug intake. Control analyses of systolic and diastolic blood pressure confirmed that drug groups did not differ in noradrenergic arousal during stimulus presentation, neither before nor immediately after free recall testing, and that yohimbine increased noradrenergic arousal only ∼30 min after completing free recall testing (Krenz et al., 2021). Moreover, drug groups did not show whole-brain differences nor differences in anterior MTL activity during stimulus presentation and, furthermore, demonstrated comparable performance in the free recall test. Since the drug was not yet effective during stimulus presentation and immediate free recall, we collapsed data across the placebo and yohimbine groups for the present analysis.

Participants performed three consecutive runs in the MRI scanner. In each run, participants were presented with the same 30 emotionally negative and 30 neutral images of scenes taken from the International Affective Picture System (Lang et al., 2008) and open Internet platforms. Emotionally negative and neutral images were matched in terms of subjectively perceived visual complexity and the number of images depicting humans or animals. Within runs, images were presented in a fully random order using MATLAB (The MathWorks) with the Psychophysics Toolbox extensions (Brainard, 1997), i.e., each image was presented once in each of the three encoding runs. On each trial, an image was presented for 3 s followed by a jittered fixation period of 4 ± 1 s. Participants were instructed to memorize the presented images and informed that there will be a subsequent memory test immediately afterward. To make sure that participants remained fully attentive throughout the task, they were instructed to press a button as soon as the fixation cross appeared on the screen.

Immediately after the repetition task, participants completed a free recall task outside the MRI scanner. Here, participants were given 15 min to recall as many of the previously viewed stimuli in as much detail as possible, while an audio recording was made. Based on these recordings, three independent raters classified an item as “remembered” if it was clearly identifiable from the participant's description. These ratings showed a very high inter-rater reliability (Light's *κ* = 0.983; *z* = 59.6; *p* < 0.001) and were thus collapsed for subsequent analyses. In case of disagreement among the raters, the majority of decision was used to determine the binary variable “subsequent memory.” Importantly, all stimuli that were presented included a different semantic gist, allowing clear identification of the recalled item.

In order to validate the emotionality of the presented images, the same participants rated each stimulus with respect to its valence and arousal on a scale from 0 (“very negative”/“not arousing”) to 10 (“very positive”/“very arousing”) on a separate day (Krenz et al., 2021, 2023), outside the MRI. In retrospect, these ratings conﬁrmed that negative images (M = 2.408; SEM = 0.078) were perceived as significantly more negative than neutral ones (M = 5.872; SEM = 0.091; paired *t* test, *t*_(102)_ = 23.367; *p* < 0.001; Cohen’s *d* = 4.039; CI[3.008, 5.071]). Furthermore, negative images (M = 5.950; SEM = 0.135) were associated with significantly higher subjective arousal than neutral ones (M = 2.759; SEM = 0.140; paired *t* test, *t*_(102)_ = 21.676; *p* < 0.001; Cohen’s *d* = 2.81; CI[1.887, 2.675]).

### Behavioral data analysis

To control for attentiveness during stimulus presentation and potential influences on subsequent memory effects, we analyzed missed responses to the fixation cross by means of a binomial generalized linear-mixed model (LMM) with the fixed effects of run (Run 1 vs Run 2 vs Run 3), subsequent memory (forgotten vs remembered), and emotion (neutral vs negative) with a random intercept for participants. The difference in subsequent memory performance depending on stimulus emotionality was analyzed by means of a paired *t* test.

All statistical analyses were performed with R Version 4.2.2 (https://www.r-project.org/) in RStudio Version 2022.12 (Posit team, 2022). All reported *p* values are two-tailed with an α-level of 0.050. Mixed model’s post hoc tests (*z-*contrasts) were applied by contrasting estimated marginal means (EMMs) of respective conditions and corrected for multiple comparisons by controlling for the false discovery rate (FDR; Benjamini, 2010) using the R package emmeans Version 1.7.2 (Lenth et al., 2018).

### MRI acquisition

MRI data were acquired using a 3 T Prisma Scanner (Siemens) with a 64-channel head coil. Each MRI session consisted of three functional runs and a magnetic (B0) field map to unwarp the functional images (TR, 634 ms; TE_1_, 4.92 ms; TE_2_, 7.38 ms; 40 slices; voxel size, 2.9 × 2.9 × 3.0 mm^3^; FOV, 224 mm). For the functional scans, T2*-weighted echo planar imaging sequences were used to obtain 2-mm-thick transversal slices (TR, 2,000 ms; TE, 30 ms; flip angle, 60^°^; FOV, 224). Additionally, a high-resolution T1–weighted anatomical image (TR, 2,500 ms; TE, 2.12 ms; 256 slices; voxel size, 0.8 × 0.8 × 0.9 mm^3^) was collected.

### Preprocessing

All scans underwent the same preprocessing steps using SPM12 (Wellcome Trust Centre for Neuroimaging). To allow for magnetic field (T1) equilibration, we discarded the first three functional scans. The images were first realigned and unwarped using the field maps and then coregistered to the structural image followed by a normalization to Montreal Neurological Institute space, as implemented in SPM12 (IXI549Space). No smoothing was performed on the echoplanar imaging data that entered the generalized lined model (GLM) for univariate and multivariate analyses.

### First-level modeling

First-level modeling was applied using SPM12 (Wellcome Trust Centre for Neuroimaging). Here, each of the 180 trials of the repetition task was modeled as an individual regressor convolved with a hemodynamic response function along with three run constants in one GLM per subject. A high-pass ﬁlter of 128 s was used to remove low-frequency drifts, and serial correlations in the time series were accounted for using an autoregressive AR(1)-model.

### Single-trial ROI–based analyses

We utilized a single-trial region of interest (ROI)-based analysis approach. Unlike the traditional condition-level, voxelwise method, this approach allows for examining the changes in activation (patterns) for each individual stimulus across repeated presentations, accounting for its emotionality and subsequent memory, within predefined brain regions.

We expected that subsequent memory for negative items would be specifically associated with an increased BOLD response in the anterior MTL, i.e., amygdala (McGaugh, 2004; Murty et al., 2011) and anterior hippocampus (Murty et al., 2011; Dandolo and Schwabe, 2018; Cowan et al., 2021), during initial exposure (Run 1). To assess whether observed effects were indeed specific to the anterior part of the MTL, we additionally examined the mid and posterior hippocampus. Anatomical masks of the anterior, mid, and posterior hippocampus (left and right) were derived using the Wake Forest University PickAtlas (Lancaster et al., 2000; Maldjian et al., 2003). Anatomical masks for the amygdala (left and right) were derived from the Harvard–Oxford Atlas as included in the FMRIB Software Library (https://fsl.fmrib.ox.ac.uk/fsl/fslwiki/FSL) with a probability threshold of 50%, reduced by overlap for the anterior hippocampus mask. Anatomical masks for additional explorative analyses in the (left and right) perirhinal and parahippocampal cortices were derived from https://neurovault.org/collections/3731/ (Ritchey et al., 2015) and reduced by overlap with the amygdala and hippocampal masks.

To investigate dynamics of emotional enhancement of memory for recurring events in the neocortex, we divided the cortex into 200 fine-grained regions using a well-established cortical parcellation scheme (Schaefer et al., 2018). While harnessing the benefits of traditional ROI analyses, i.e., increasing statistical power and interpretability by focusing on functionally homogeneous regions rather than individual voxels, this parcellation-based approach captures the entirety of the neocortex. This allows a comprehensive examination of activity across encoding runs throughout the neocortex and, thus, along with correcting for multiple comparisons, addresses the complexity of neocortical encoding-related activity more effectively than a limited set of predefined ROIs (Cooper and Ritchey, 2020).

Both univariate and multivariate data were analyzed by means of LMMs, fitted using restricted maximum likelihood with a variable intercept for subjects and items in R. In cases of singular fits or convergence issues, where the variance–covariance matrix of the random effects approaches noninvertibility or the optimization algorithm does not find optimal parameter estimates, respectively, the respective model was refitted after excluding the random effect causing these estimation difficulties, ensuring a more stable and interpretable fit (Matuschek et al., 2017). Each fixed effect was tested against zero using Satterthwaite’s approximation method, which provides reliable estimates of the degrees of freedom in LMMs while minimizing Type 1 error rates (Luke, 2017). To account for multiple comparisons, *p* values were FDR (Benjamini, 2010) corrected, accounting for the number of ROIs in each analysis. To further ensure the reliability of our results, data were resampled with replacement to create 10,000 simulated datasets as implemented in lme4 (Bates et al., 2015), incorporating a seed value for reproducibility. From the distribution of these simulated estimates, we computed 95% confidence intervals, representing a robust range of plausible effect values. Both bootstrapped confidence intervals and FDR-corrected *p* values were taken into account when assessing the statistical significance of an effect at a two-sided α–level of 0.050, aligning with previous recommendations to consider multiple estimates as evidence for an effect (Cumming, 2014; Valentine et al., 2019). To keep the results of our neocortical analyses consistent with common practices in whole-brain fMRI analyses, adjacent significant ROIs with a similar effect pattern, i.e., showing an emotion-specific increase or decrease over runs in case of our univariate analyses, were arranged into clusters using custom code in Python. Specifically, we used the package nibabel (Brett et al., 2020) for handling NIfTI images, SciPy (Virtanen et al., 2020) for calculating center of mass and adjacency, and NetworkX (Hagberg et al., 2008) for clustering adjacent ROIs. In case of clustered ROIs, results of ROIs with highest absolute *t* values are reported. Results of fMRI analyses were displayed using BrainNet Viewer (Xia et al., 2013, http://www.nitrc.org/projects/bnv/), where, for visualization purposes, every voxel within a region was allocated the same *t* statistic, based on this region’s fixed effect estimation in the respective LMM.

#### Univariate analysis

For our univariate analyses, mean single-trial beta series were extracted by averaging across voxels within each ROI per participant and trial. Emotionally dependent activity changes over presentation runs in single-trial betas were analyzed by means of trial-wise LMMs with subsequent memory (forgotten vs remembered), emotion (neutral vs negative), a linear increase predictor over encoding runs, centered around Run 2, and their interactions as fixed effects. We report results for all ROIs that showed a significant memory × emotion × run interaction after FDR correction in these LMMs.

To follow up on these three-way interaction effects, we applied post hoc analyses on the model’s EMMs and slope coefficients (estimated marginal slopes, EMSs) contrasting remembered > forgotten items. Here, EMM and EMS represent the predicted activity at each level of emotion and run and the change in activity over repeated presentations for each level of emotion, respectively, while adjusting for the level of each predictor, capturing emotion-specific changes across repetitions associated with subsequent memory. To account for multiple comparisons in our post hoc analyses, *p* values of resulting contrast estimates were further FDR-corrected (Benjamini, 2010), as implemented in the R package emmeans (Lenth et al., 2018). Due to the high number of degrees of freedom in these univariate analyses, we furthermore applied a permutation procedure to the resulting, significant model estimates allowing for an assessment of the significance under the null hypothesis that does not depend on the degrees of freedom of the model. For this, trial labels within participants were shuffled over 1,000 iterations to generate a null distribution for each effect to compare the observed *t* statistic from each model against this distribution. Importantly, this procedure confirmed all reported results (memory × emotion × run, all *p*_perm_ < 0.022), demonstrating that these findings are robust and not attributed to the high degrees of freedom in the model.

#### Multivariate analysis

While our univariate analyses allowed investigating activity changes over repeated exposures underlying emotional enhancement of memory, we further applied multivariate pattern analyses probing stable (vs variable) activation patterns over repeated exposures underlying successful emotional memory formation. For this, single-trial first–level betas were transformed into *t* statistics to increase the reliability of the measured activation patterns by normalizing for noise (Walther et al., 2016). The data were then subjected to representational similarity analyses (Kriegeskorte et al., 2008) using custom scripts in MATLAB (The MathWorks). Specifically, we computed item-wise encoding pattern similarity (Xue et al., 2010, 2013; Feng et al., 2019) within each ROI and subject, by correlating activation patterns of an item in a specific run with activation patterns of the same item in a subsequent run (Fig. 4*a*). The resulting Pearson’s *r* values were further Fisher *z*-transformed and averaged over run comparisons before statistical analyses in R were conducted. Fisher *z*-transformed *r* values were analyzed by means of item-wise LMMs with the factors subsequent memory (forgotten vs remembered), emotion (neutral vs negative), and their interactions as fixed effects and random intercepts of subjects and items. We report results for all ROIs that showed a significant memory × emotion interaction after FDR correction in these LMMs. Post hoc analyses were conducted by computing the model’s EMMs contrasting remembered > forgotten items. Resulting EMMs thus represent activation pattern similarity associated with subsequent memory for emotionally negative or neutral items.

Previous work suggests that successful memory encoding is associated with stable pattern representations during repeated presentations as an indicator of a consistent reactivation of neural activations across repetitions (Xue et al., 2010, 2013; Feng et al., 2019). Thus, we expected pattern similarity across repetitions to be positively associated with subsequent memory, and that pattern similarity for subsequently remembered items should be increased for emotionally negative items, particularly in frontoparietal neocortical regions associated with attentional and cognitive control processes which may enhance immediate emotional memory enhancement (Talmi, 2013). As we expected the anterior MTL to be primarily involved in the initial, yet transient, enhancement of emotional memory formation, we did not expect stable activation pattern representations in this region.

We further explored whether regions associated with more stable activation patterns for subsequently remembered emotional items in the above analysis support encoding of recurrent emotional events through item-specific reinstatement across repetitions or more general processes associated with successful emotional memory encoding, such as stable attention to encoding material. To investigate this, we contrasted within-item similarity (the similarity between activation patterns of the same item across different runs) with between-item similarity (the similarity between activation patterns of different items within the same subsequent memory and emotion category). This analysis was conducted using LMMs with the factors emotion (neutral vs negative), subsequent memory (forgotten vs remembered), and item specificity (between-item vs within-item similarity). In these models, a significant, positive memory × emotion × item specificity interaction indicates a significantly higher association between within-item compared with between-item pattern similarity and subsequent emotional (vs neutral) memory and, thus, item-specific pattern reinstatement across repeated encounters of emotional events.

#### Multilevel-moderated mediation analysis

In a final step, we analyzed whether initial MTL engagement may modulate neocortical pattern stability linked to emotional memory enhancement. Given the hierarchical structure of our data—with items nested in subjects—we employed a multilevel-moderated mediation approach utilizing the R packages mediation (Tingley et al., 2014) and lme4 (Bates et al., 2015). This entailed fitting two models: (1) an LMM estimating the mediator (encoding pattern similarity in Fisher-transformed *r*) as a function of the predictor (emotion, neutral vs negative), the moderator (anterior MTL activity), their interaction, and the random intercept of participants and (2) a generalized LMM predicting the outcome variable (subsequent memory, forgotten vs remembered), based on the interaction between emotion (neutral vs negative) and anterior MTL activity, the interaction between encoding pattern similarity and anterior MTL activity, and the random intercept of participants. Continuous variables were subject–mean centered before entering the models as predictors. Mediator and outcome model fits were then combined to estimate the indirect effect, i.e., influence of emotion on subsequent memory via encoding pattern similarity (Fig. 5, $a_{1}\times b$), and the direct effect of emotion on subsequent memory when controlling for both the mediator and moderator $\left(c_{1}^{\prime }\right)$. For this, a quasi-Bayesian Monte Carlo method with a total of 5,000 simulations including a predefined seed was applied, offering a robust estimation of the direct effect by approximating the distribution of the indirect effect (Tingley et al., 2014). This approach allows evaluating conditional indirect and direct effects under different levels of the moderator (Tingley et al., 2014). In this analysis, we specifically focused on the modulating role of univariate activation in the left amygdala and right anterior hippocampus at Run 1, which our univariate analyses identified as highly impactful in the successful encoding of emotional memories (Fig. 2), and the mediating role of stable activation patterns in regions associated with emotional memory enhancement as identified in our multivariate analysis [orbitofrontal cortex (OFC), anterior cingulate cortex (ACC), superior temporal sulcus (STS), superior parietal lobule (SPL), postcentral sulcus; Fig. 4*b*]. Thus, we fitted one moderator and one outcome model per anterior MTL and encoding stability ROI and corrected for multiple comparisons by applying FDR correction, taking into account the number of ROIs per analysis. To follow up on a significant indirect effect (FDR-corrected for the total number of ROIs) and a numeric change in the indirect effect from low (−1 SD) over average to high (+1 SD) levels of the moderator and a significant indirect effect for at least one of the moderator levels, we further estimated the index of moderated mediation (Hayes, 2015). In the context of a moderated mediation, the indirect effect ω of a predictor on an outcome through a mediator can be expressed as $\omega =\;a_{1}b\;+\;a_{2}bW$, where the intercept $a_{1}b$ represents the indirect effect when the moderator (*W*) equals zero and the slope $a_{2}b$ quantifies the change in the indirect effect as a linear function of the moderator, i.e., the index of moderated mediation (Hayes, 2015). Thus, the index of moderated mediation is reflected by the product of the moderated effect of the predictor on the mediator (emotion × anterior MTL activity on encoding similarity; $a_{2}$) and the mediator’s effect on the outcome when controlling for the effect of the predictor (main effect encoding similarity on subsequent memory; b). For rigorous statistical inference, we applied bias-corrected bootstrap confidence intervals, drawing 10,000 samples from the original dataset to construct a reliable distribution of the index of moderated mediation, following previous recommendations (Hayes, 2015).

### Data accessibility

The behavioral and fMRI data generated in this study are provided at http://doi.org/10.25592/uhhfdm.13783.

### Code accessibility

Custom code used to model and analyze the data is available at https://zenodo.org/doi/10.5281/zenodo.10210565.

## Results

### Emotional enhancement of immediate free recall

**Figure 1.**
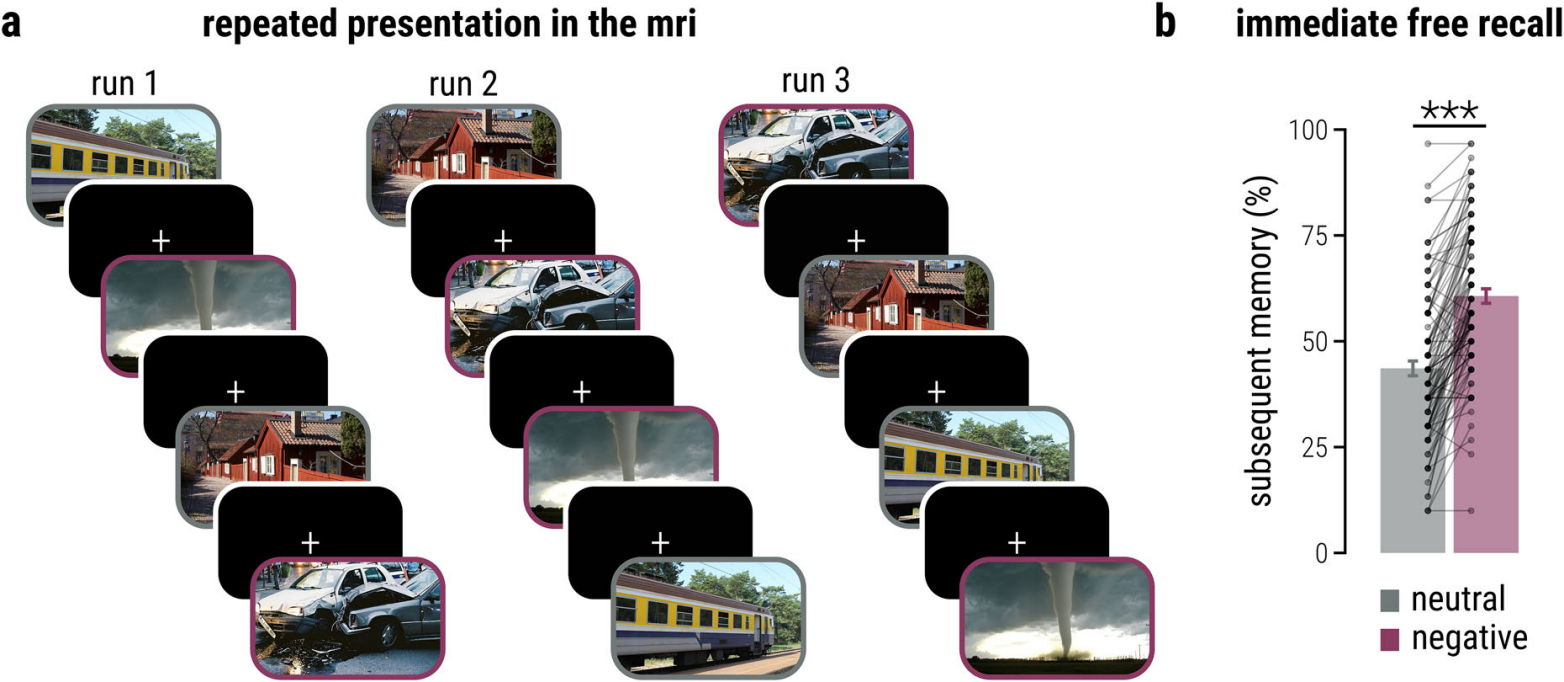
Experimental task and subsequent free recall performance. ***a***, Participants repeatedly viewed images that were either emotionally neutral or emotionally negative in three consecutive runs in an MRI scanner. All included images are licensed under Creative Commons BY-SA license: image of train is courtesy of John Samuel (https://bit.ly/3SkVjma; changed), image of tornado is courtesy of Justin Hobson (https://bit.ly/3SbJTkK; changed), image of buildings is courtesy of Holger Ellgaard (https://tinyurl.com/4cybwv2f; changed), image of car crash is courtesy of Dino Kužnik (https://bit.ly/3SodgAx; changed). ***b***, Shortly after repeated presentation, participants were asked to freely recall all previously presented images. Subsequent memory was significantly higher for emotionally negative compared with neutral items (two-tailed paired *t* test, *p* < 0.001; *n* = 103). Connected dots represent the percentage of remembered items per participant and emotion; bars represent mean percentage of remembered items per emotion ± SEM. ***p < 0.001.

To elucidate the neural evolution of emotional memory enhancement across repeated encoding sessions, 103 participants saw 30 emotionally negative and 30 neutral scene images across three consecutive runs in the MRI scanner (Fig. 1*a*). To control for attentiveness during encoding, participants were asked to respond with a button press as soon as a fixation cross appeared on the screen. On average, participants missed responding to only 0.809% (SEM = 0.176%) of the fixation crosses, indicating an overall high attentiveness during the encoding task. While missed responses increased over runs (main effect run, *β* = 0.540, CI[0.164, 0.914], *z* = 2.816, *p* < 0.001), the overall number of missed trials per run remained very low throughout the task (Run 1, M = 0.485%; SEM = 0.114%; Run 2, M = 0.841%; SEM = 0.271%; Run 3, M = 1.100%; SEM = 0.286%). Moreover, misses and, by implication, participant’s attentiveness immediately before stimulus presentation did not differ between subsequently remembered and forgotten trials (all |*β*| < 1.613; all *p* > 0.107) nor between emotionally negative and neutral images (all |*β*| < 1.051; all *p* > 0.293). In the free recall test immediately after repeated stimulus presentation, participants recalled significantly more negative (M = 60.680%; SEM = 1.708%) than neutral items (M = 43.592%; SEM = 1.743%; paired *t* test, *t*_(102)_ = 13.736; *p* < 0.001; Cohen’s *d* = 0.976; CI[0.805, 1.146]; Fig. 1*b*), thus demonstrating the well-known emotional enhancement of immediate memory (Talmi et al., 2007; Murty et al., 2011; Talmi and McGarry, 2012; Schümann et al., 2018).

### Trajectory of anterior MTL involvement across repetitions distinguishes memory formation of emotional and neutral events

To examine the neural mechanisms involved in the evolution of memory for recurring emotional events, we analyzed activation changes over multiple presentation runs for both emotionally negative and neutral scene images, taking into account subsequent memory of the specific item. We expected that subsequently enhanced memory for negative compared with neutral items would be associated with activity in the anterior MTL, specifically the amygdala (McGaugh, 2004; Murty et al., 2011; Dahlgren et al., 2020) and the anterior portion of the hippocampus (Murty et al., 2011) during the first exposure to an emotionally negative item, i.e., in Run 1 (Ranganath and Rainer, 2003; Cowan et al., 2021).

**Figure 2.**
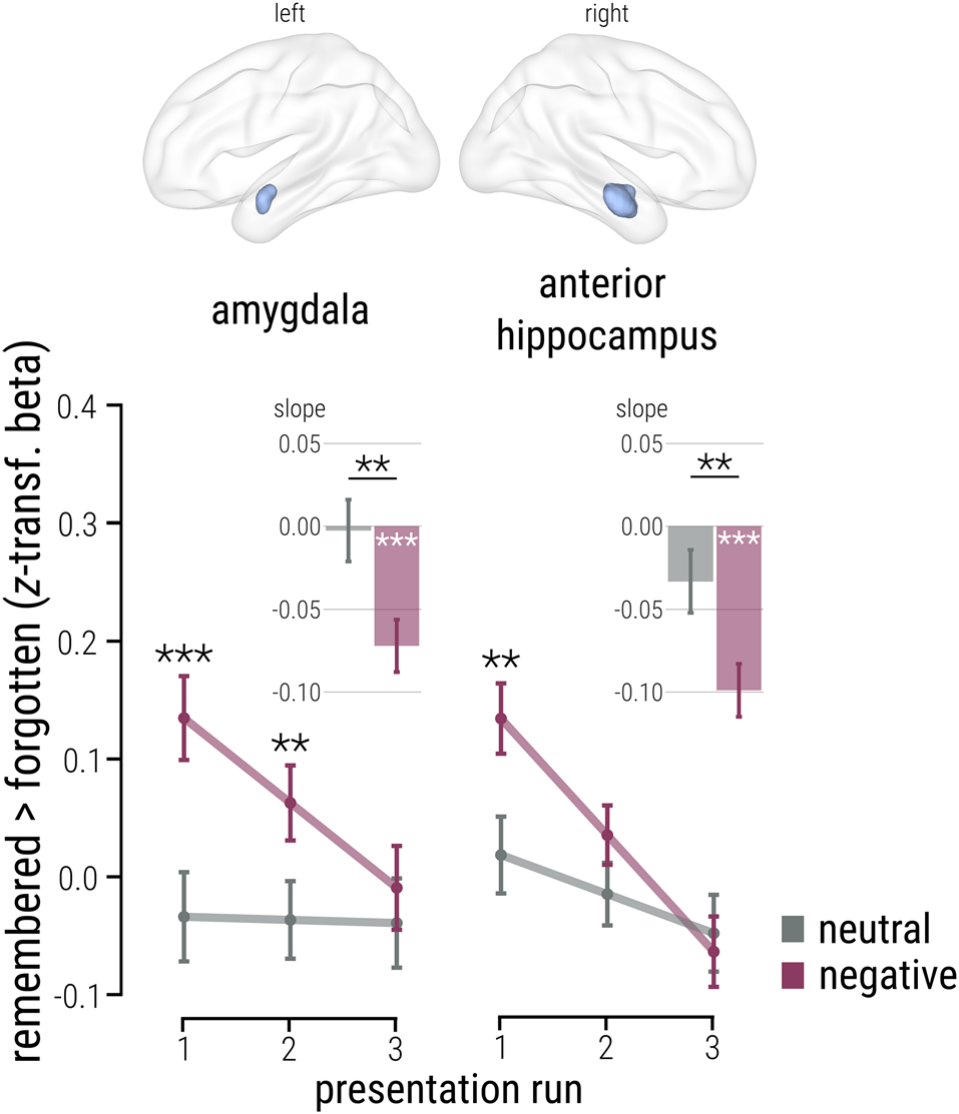
Transient anterior MTL involvement linked to subsequent memory for recurring emotional events. While left amygdala and right anterior hippocampus activity at initial presentation was associated with subsequent memory of emotional (vs neutral) events, anterior MTL involvement significantly decreased over repetitions (LMMs, memory × emotion × run, both *p*_corr_ = 0.040). During the final presentation run, no association between subsequent emotional (vs neutral) memory and anterior MTL activation was evident. These findings indicate that the emotional enhancement of memory is linked to rapid anterior MTL recruitment when an emotional stimulus is encountered for the first time and that anterior MTL contribution decreases over repeated exposures. Note that line and bar plots show conventional (dependent) post hoc tests to follow up the significant three-way interaction in the left amygdala and right anterior hippocampus. Connected dots depict the EMMs for the contrast “remembered > forgotten” per level of emotion and run ± SE; bars represent EMSs for the same contrast per level of emotion ± SE. All *n* = 103. Reported *p* values are two-tailed and FDR-corrected for multiple comparisons while accounting for number of regions of interest in this analysis (*p*_corr_). ***p < 0.001; **p < 0.010.

Analyzing BOLD responses on a single-trial level by means of an LMM with a predictor modeling a linear increase over presentation runs and the factors emotion (neutral vs negative) and subsequent memory (forgotten vs remembered), we found a significant memory × emotion × run interaction in the left amygdala (*β* = −0.090; CI[−0.156, −0.018]; *t*_(18350.200)_ = −2.478; *p*_corr_ = 0.040; Fig. 2, left panel). In the right amygdala, there was no such interaction (*p*_corr_ = 0.241), nor were there other significant effects involving memory in this region (all *p*_corr_ > 0.318). For the anterior hippocampus, the same significant memory × emotion × run interaction was significant in the right hemisphere (*β* = −0.080; CI[−0.156, −0.012]; *t*_(18363.510)_ = −2.328; *p*_corr_ = 0.040; Fig. 2, right panel), whereas no such interaction was detected in the left anterior hippocampus (*p*_corr_ = 0.256). Similarly, no other effects involving memory reached significance in the left anterior hippocampus (all *p*_corr_ > 0.318).

To disentangle this three-way interaction in the left amygdala and right anterior hippocampus, we applied post hoc analyses on the model’s EMMs and slope coefficients (EMSs) contrasting remembered > forgotten items, as visualized in Figure 2. Here, EMM and EMS values represent the predicted anterior MTL activity at each level of emotion (neutral vs negative) and run (Run 1 vs Run 2 vs Run 3) and the change in anterior MTL activity over repeated encounters for each level of emotion, respectively, capturing emotion-specific changes across repetitions in anterior MTL activity associated with successful memory encoding. These analyses demonstrated that subsequently remembered emotional items were associated with a significantly higher anterior MTL activity compared with neutral items during initial exposure, i.e., Run 1 (interaction contrasts; left amygdala, EMM = 0.169; CI[0.084, 0.253]; *t*_(182.172)_ = 3.933; *p* < 0.001; see line plot in Figure 2, left panel; right anterior hippocampus, EMM = 0.116; CI[0.044, 0.188]; *t*_(376.749)_ = 3.158; *p* = 0.005; Fig. 2, line plot, right panel). Anterior MTL activity, however, did not significantly differ between emotionally negative and neutral images at the last exposure, i.e., Run 3 (all |EMM| < 0.030; all *p* > 0.486; Fig. 2). Accordingly, both anterior MTL regions showed a significant decrease in activity for emotionally negative items (paired contrasts; left amygdala, EMS = −0.072; CI[−0.108, −0.037]; *t*_(18350.218)_ = −4.547; *p* < 0.001; right anterior hippocampus, EMS = −0.099; CI[−0.135, −0.063]; *t*_(18363.538)_ = −6.178; *p* < 0.001), and this decrease was significantly larger compared with neutral items (interaction contrasts; left amygdala, EMS = −0.069; CI[−0.117, −0.021]; *t*_(18350.215)_ = −2.832; *p* = 0.005; Fig. 2, bar plot, left panel; right anterior hippocampus, EMS = −0.066; CI[−0.114, −0.017]; *t*_(18363.516)_ = −2.654; *p* = 0.008; Fig. 2, bar plot, right panel). No significant change in anterior MTL activity over runs was observed for neutral items (paired contrasts, all |EMS| < 0.034; all *p* > 0.078; Fig. 2, bar plots). These findings indicate that emotional enhancement of immediate memory is linked to increased anterior MTL recruitment specifically during the first exposure to an emotional item. With repeated exposure, anterior MTL involvement decreased. For neutral items that were subsequently remembered, anterior MTL activity remained rather stable across repeated item presentations.

To assess whether our findings are specific to the anterior part of the MTL, we additionally analyzed activity in the mid and posterior part of the hippocampus, again by means of LMMs with a predictor modeling a linear increase over runs and the factors subsequent memory (forgotten vs remembered), emotion (neutral vs negative), and their interaction. This analysis indicated that bilateral posterior hippocampal activity was overall positively associated with successful memory formation (paired contrasts; left, EMM = 0.044; CI[0.074, 0.013]; *t*_(7455.599)_ = 2.826; *p* = 0.005; right, EMM = 0.039; CI[0.07, 0.009]; *t*_(6878.434)_ = 2.551; *p* = 0.011; main effect memory, left, *β* = 0.047; CI[0.006, 0.089]; *t*_(11249.719)_ = 2.245; *p*_corr_ = 0.0496; right, *β* = 0.053; CI[0.012, 0.095]; *t*_(10643.403)_ = 2.512; *p*_corr_ = 0.048), yet did not differ between emotional and neutral items (memory × emotion, all |*β*| < 0.028; all *p*_corr_ > 0.796) and did not significantly change over runs (memory × run, all |*β*| < 0.045; all *p*_corr_ > 0.315; memory × emotion × run, all |*β*| < 0.021; all *p*_corr_ > 0.749). No effects were significant in the mid part of the MTL (all |*β*| < 0.059; all *p*_corr_ > 0.151).

Given previous literature (Yonelinas and Ritchey, 2015; Ritchey et al., 2019) associating perirhinal and parahippocampal cortices with emotional memory formation, we furthermore explored the trajectory of the involvement of these regions across repetitions of emotional (vs neutral) events. The right perirhinal cortex (PRC) showed a similar trend to the anterior MTL, with a higher involvement for successfully remembered emotionally negative compared with neutral items at encoding run 1 (EMM = 0.159; CI[0.081, 0.236]; *t*_(311.451)_ = 4.039; *p* < 0.001), which decreased over encoding runs (EMS = −0.104; CI[−0.141, −0.066]; *t*_(18371.03)_ = −6.25; *p* < 0.001) but failed to reach statistical significance (memory × emotion × run, *β* = −0.088; *t*_(18371.030)_ =−2.382; *p*_corr_ = 0.068). No effects including the factor memory were significant in the left PRC (all |*β*| < 1.675; all *p*_corr_ > 0.130). For the right parahippocampal cortex (PHC), we observed a significant main effect of memory (*β* = 0.060; *t*_(14220.64)_ = 2.964; *p*_corr_ = 0.012), indicating an involvement of this region in successful memory encoding, with a similar trend for the left PHC (*β* = 0.041; *t*_(16293.33)_ = 1.974; *p*_corr_ = 0.068). No interaction involving the factor memory approached significance in both the right (all |*β*| < 1.654; all *p*_corr_ > 0.130) and left PHC (all |*β*| < 0.449; all *p*_corr_ > 0.653).

### Opposite trajectory of anterior and posterior neocortical involvement across repetitions for emotional and neutral events

In a next step, we investigated the trajectory of neocortical involvement during repeated exposure to emotionally negative and neutral images by segmenting the cortex into 200 fine-grained parcellations (Schaefer et al., 2018). By focusing on functionally homogeneous regions rather than individual voxels or a limited set of neocortical ROIs and applying correcting for multiple comparisons, this method allows a comprehensive analysis of activation trajectories across the entire neocortex during recurring emotional events. Trial-wise BOLD responses were analyzed by means of an LMM with a predictor modeling a linear increase over presentation runs and the factors emotion (neutral vs negative) and subsequent memory (forgotten vs remembered). All ROIs that exhibited a significant memory × emotion × run interaction after FDR correction are further reported and subjected to post hoc analyses.

**Figure 3.**
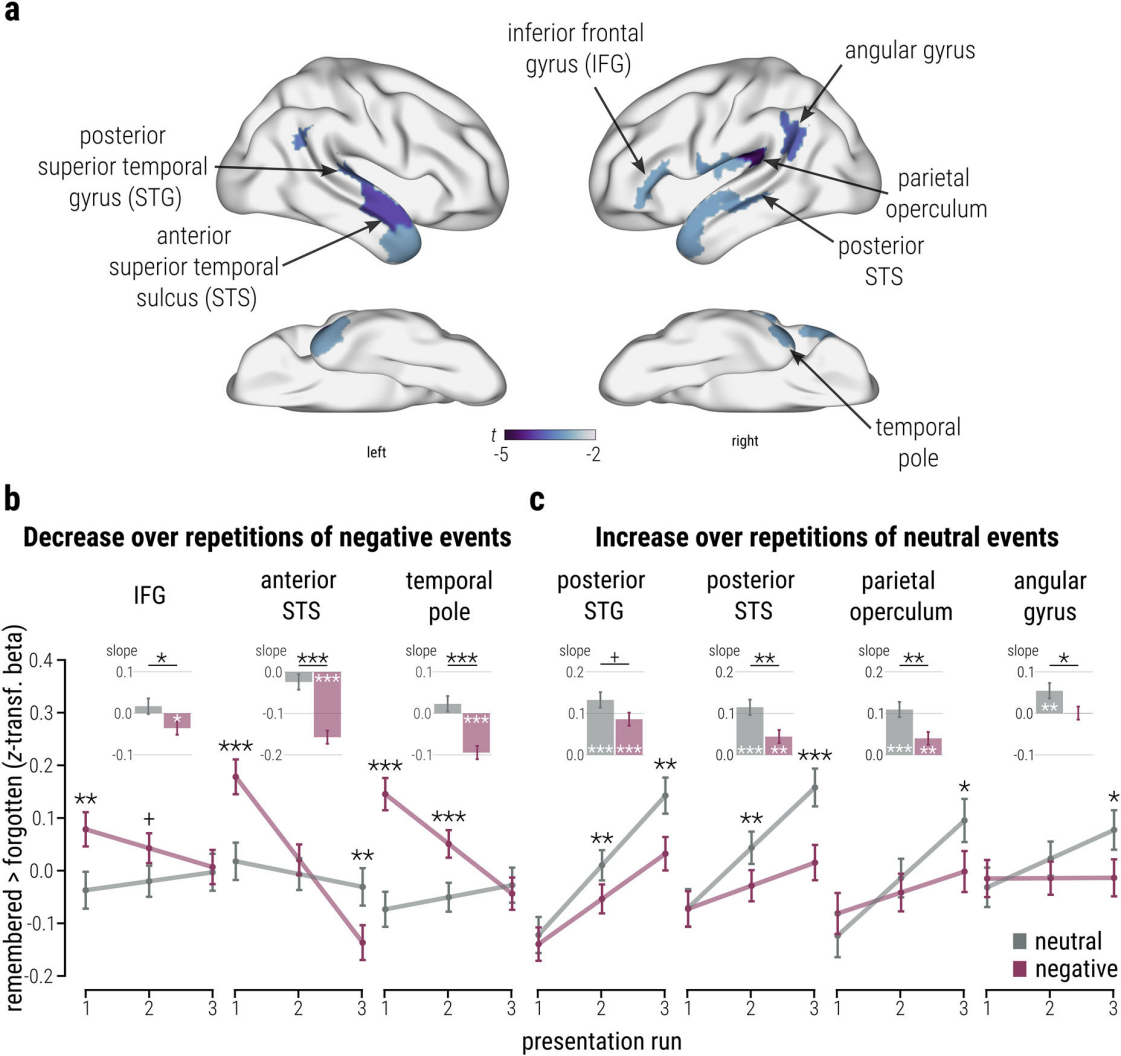
Differential neocortical trajectories for repeated exposure to emotional and neutral events. ***a***, ROIs indicating a significant three-way interaction in LMMs with the factors subsequent memory, emotion, and presentation runs. Surface maps depict the *t* statistic for the fixed effect estimate for the memory × emotion × run interaction in each ROI, with all voxels within a ROI assigned the same value for visualization purposes. ***b***, Post hoc analyses revealed that bilateral anterior temporofrontal activity during initial exposure was associated with subsequent memory for emotional (vs neutral) events, with decreasing involvement across presentation runs (LMMs, memory × emotion × run, right IFG, *p*_corr_ = 0.047; left anterior STS, *p*_corr_ = 0.008; right temporal pole, *p*_corr_ = 0.047). ***c***, Conversely, posterior temporal and parietal cortices were associated with subsequent memory after several exposures, specifically for emotionally neutral events (LMMs, right posterior STG, *p*_corr_ = 0.021; right posterior STS, *p*_corr_ = 0.039; right parietal operculum, *p*_corr_ = 0.001; right angular gyrus, *p*_corr_ = 0.021). These findings highlight the rapid, yet transient engagement of anterior temporofrontal cortices in emotional memory formation, whereas subsequent memory for neutral events may require multiple exposures and rely on posterior neocortical areas. Note that panels ***b*** and ***c*** show conventional (dependent) post hoc tests to follow up the significant interaction effect shown in panel ***a***. Connected dots depict the EMMs for the contrast “remembered > forgotten” at each level of the predictors emotion and run ± SE; bars represent EMSs for the same contrast per level of emotion ± SE. All *n* = 103. Reported *p* values are two-tailed and FDR-corrected for multiple comparisons accounting for the number of ROIs in this analysis (*p*_corr_). ***p < 0.001; **p < 0.010; **p < 0.050; ^+^*p* < 0.060.

These analyses revealed an initial enhancement of activity associated with emotional (vs neutral) memory formation in the right inferior frontal gyrus (IFG; interaction contrast, EMM = 0.116; CI[0.040, 0.191]; *t*_(291.589)_ = 3.024; *p* = 0.008; memory × emotion × run, *β* = −0.110; CI[−0.169, −0.038]; *t*_(18340.610)_ = −2.972; *p*_corr_ = 0.047) and bilateral anterior temporal cortices (interaction contrasts; peak in left anterior STS, EMM = 0.160; CI[0.088, 0.233]; *t*_(343.210)_ = 4.352; *p* < 0.001; peak in right temporal pole, EMM = 0.219; CI[0.148, 0.29]; *t*_(400.730)_ = 6.043; *p* < 0.001; memory × emotion × run, left, *β* = −0.140; CI[−0.207, −0.073]; *t*_(18330.090)_ = −3.940; *p* = 0.008; right, *β* = −0.110; CI[−0.174, −0.042]; *t*_(18340.840)_ = −3.012; *p*_corr_ = 0.047; Fig. 3*a*,*b*, line plots). With repeated exposure, activity associated with subsequent emotional memory decreased in both anterior temporal cortices (left, EMS = −0.157; CI[−0.193, −0.122]; *t*_(18330.126)_ = −9.941; *p* < 0.001; right, EMS = −0.095; CI[−0.130, −0.059]; *t*_(18340.890)_ = −5.916; *p* < 0.001) and the IFG (EMS = −0.036; CI[−0.072, −0.0001]; *t*_(18340.631)_= −2.247; *p* = 0.049), whereas no significant activity changes over runs were observed for neutral items (all |EMS| < 0.025; all *p* > 0.193; paired contrasts; Fig. 3*b*, bar plots). Contrasting the activity change over runs for emotional and neutral items confirmed that subsequent emotional compared with neutral memory was associated with a significantly higher decrease over repetitions in both anterior temporal cortices (left, EMS = −0.133; CI[−0.181, −0.085]; *t*_(18330.071)_ = −5.436; *p* < 0.001; right, EMS = −0.117; CI[−0.166, −0.069]; *t*_(18340.82)_ = −4.755; *p* < 0.001) and the IFG (EMS = −0.053; CI[−0.101, −0.005]; *t*_(18340.614)_ = −2.146, *p* = 0.032; interaction contrasts; see bar plots in Fig. 3*b*). Consequently, at the time of the final presentation run, BOLD responses associated with subsequent memory in the IFG (EMM = 0.010; *p* = 0.796) and right anterior temporal lobe (EMM = −0.016; *p* = 0.655) did not significantly differ between emotional and neutral items, while it was even reduced for emotionally negative compared with neutral items in the left anterior temporal cortex (EMM = −0.106; CI[−0.178, −0.033]; *t*_(344.068)_ = −2.867; *p* = 0.007; interaction contrasts; Fig. 3*b*, line plots). Notably, both the anterior temporal lobe and right IFG have previously been recognized for their pivotal role in memory processes, particularly in semantic control (Patterson et al., 2007; Ralph et al., 2017) and emotional enhancement of memory (Dolcos et al., 2004; Ritchey et al., 2011; Weintraub-Brevda and Chua, 2018), respectively. Our findings point to a transient role of these anterior temporofrontal regions in supporting emotional memory formation, with a heightened BOLD response for emotional events, specifically during initial exposure, mirroring our results in the anterior MTL. A midline cluster (not depicted) showed a similar pattern of results, with a significantly higher decrease in activity over runs for recurrent emotional (peak in the mid cingulate cortex, paired contrast, EMS = −0.035; CI[−0.071, −0.0001]; *t*_(18334.427)_ = −2.25; *p* = 0.049) compared with neutral items (paired contrast: EMM = 0.0236; *p* = 0.203; interaction contrast, EMS = −0.059; CI[−0.107, −0.011]; *t*_(18334.391)_ = −2.427; *p* = 0.015; memory × emotion × run, *β* = −0.120; CI[−0.188, −0.051]; *t*_(18334.46)_ = −3.37; *p*_corr_ = 0.026), however, without significant activity differences between negative and neutral items at Run 1 (EMM = 0.062; *p* = 0.128), Run 2 (EMM = 0.003; *p* = 0.900), and Run 3 (EMM = −0.056; *p* = 0.128).

While these data indicated that anterior neocortical regions were more strongly engaged during the initial encounter of emotional events, with this engagement decreasing across repeated exposures, posterior neocortical areas exhibited a markedly different pattern. In the posterior temporal cortex, there was no significant difference in activation between emotionally negative and neutral items at initial exposure (Run 1; peak in the left posterior superior temporal gyrus, STG, EMM = −0.017; *p* = 0.602; peak in the right posterior STS, EMM = −0.002; *p* = 0.964; interaction contrasts; Fig. 3*c*, line plots). However, at final exposure (Run 3), both posterior temporal cortices showed a marked increase for neutral compared with negative items (interaction contrasts; left, EMM = −0.110; CI[−0.175, −0.045]; *t*_(718.708)_ = −3.330; *p* = 0.003; right, EMM = −0.143; CI[−0.211, −0.075]; *t*_(496.588)_ = −4.118; *p* < 0.001; memory × emotion × run; left, *β* = −0.124; CI[−0.192, −0.060]; *t*_(18321.820)_ = −3.511; *p*_corr_ = 0.021; right, *β* = −0.113; CI[−0.181, −0.046]; *t*_(18346.77)_ = −3.203; *p*_corr_ = 0.039; Fig. 3*a*,*c*, line plots). Here, successful memory formation was associated with a significant increase in activation over runs, specifically for neutral items (paired contrasts; left, EMS = 0.132; CI[0.091, 0.174]; *t*_(18321.725)_ = 7.103; *p* < 0.001; right, EMS = 0.114; CI[0.073, 0.156]; *t*_(18346.776)_ = 6.154; *p* < 0.001), and, to a significantly lesser degree (interaction contrast; right: EMS = −0.071; CI[−0.118, −0.023]; *t*_(18346.776)_ = −2.893; *p* = 0.004), for emotionally negative items (paired contrasts; left, EMS = 0.086; CI[0.050, 0.121]; *t*_(18321.77)_ = 5.43; *p* < 0.001; right, EMS = 0.044; CI[0.009, 0.079]; *t*_(18346.776)_ = 2.786; *p* = 0.005; Fig. 3*c*, bar plots). A right perisylvian cluster (peak in the parietal operculum; memory × emotion × run, *β* = −0.157; CI[−0.224, −0.091]; *t*_(18328.32)_ = −4.554; *p_corr_* = 0.001; Fig. 3*a*) showed a similar pattern of results, with successful memory encoding being associated with a significant increase over encoding runs, which was significantly higher for neutral (paired contrast, EMS = 0.109; CI[0.069, 0.150]; *t*_(18328.271)_ = 6.017; *p* < 0.001) compared with emotionally negative items (paired contrast, EMS = 0.040; CI[0.005, 0.074]; *t*_(18328.379)_ = 2.582; *p* = 0.010; interaction contrast, EMS = −0.070; CI[−0.116, −0.023]; *t*_(18328.307)_ = −2.919; *p* = 0.004; Fig. 3*c*, bar plots).

In the inferior PPC, specifically within the bilateral angular gyrus, we observed a significant activation increase over runs for neutral items (paired contrasts; left, EMS = 0.064; CI[0.022, 0.106]; *t*_(18352.785)_ = 3.433; *p* = 0.001; right, EMS = 0.054; CI[0.013, 0.096]; *t*_(18359.129)_ = 2.938; *p* = 0.007) that was significantly higher compared with negative items (interaction contrasts; left, EMM =−0.081; CI[−0.129, −0.033]; *t*_(18352.776)_ = −3.302; *p* = 0.001; right, EMS = −0.054; CI[−0.101, −0.006]; *t*_(18359.143)_ = −2.213; *p* = 0.027), which did not significantly change over repetitions (paired contrasts, all *p* > 0.290; memory × emotion × run; left, *β* = −0.123; CI[−0.190, −0.057]; *t*_(18352.820)_ = −3.473; *p*_corr_ = 0.021; right, *β* = −0.129; CI[−0.198, −0.058]; *t*_(18359.16)_ = −3.656; *p*_corr_ = 0.017; Fig. 3*a*,*c*, rightmost bar plot). Accordingly, at final presentation (Run 3), the right angular gyrus displayed a significantly higher activity for neutral items compared with emotionally negative items that were subsequently recalled (EMM = −0.091; CI[−0.162, −0.020]; *t*_(362.745)_ = −2.506; *p* = 0.038; Fig. 3*c*, rightmost line plot). Intriguingly, the angular gyrus has previously been implicated in the rapid formation of memory representations across repeated encoding (Brodt et al., 2016). Our findings thus indicate memory formation in the angular gyrus for neutral items but less so for emotionally negative items over repeated exposure.

The applied neocortical parcellation furthermore offers the advantage that each ROI is assigned to one of 17 large-scale network labels (Yeo et al., 2011; Schaefer et al., 2018), allowing us to explore whether the identified differential trajectories in subsequent memory effects for recurring emotionally negative (vs neutral) events may be linked to distinct large-scale networks. Indeed, the repetition-related decrease in subsequently remembered (vs forgotten) negative vs neutral items was predominantly associated with the Default Mode Network B (57.14% of all ROIs showing this effect), with additional contributions from the Control Network C (14.29%), the Somatomotor Network A (14.29%), and the Temporoparietal Network (14.29%). In contrast, the increase over repetitions for subsequently remembered neutral items was most strongly associated with the Somatomotor Network B (62.50%), followed by the Default Mode Network B (25.00%), and the Default Mode Network A (12.50%). Notably, the Default Mode Network B was the only network involved in both effect patterns. Considering the total number of ROIs within each network, 23.53% of ROIs in the Default Mode Network B contributed to the decrease in emotionally negative items, while 11.76% of its ROIs contributed to the increase in emotionally neutral items. For other networks, both the Control Network C and the Temporoparietal Network, each with 16.67% of network ROIs, contributed to the decrease in emotionally negative items, as did 5.26% of ROIs in the Somatomotor Network A. In contrast, 33.33% of ROIs in the Somatomotor Network B and 7.14% of ROIs in the Default Mode Network A contributed to the increase for emotionally neutral items.

Together, our univariate analyses across the neocortex suggest a differential pattern of anterior and posterior neocortical involvement over repeated exposure to emotional compared with neutral events. While anterior temporofrontal areas were associated with subsequent emotional memory at initial exposure, posterior temporal and parietal cortices were involved in subsequent neutral memory after several encoding runs. Notably, no region showed an initial prioritization of emotionally neutral over negative memories or significantly higher activation for emotionally negative items during final exposure.

### Subsequent emotional memory is associated with stable neocortical pattern representations across repetitions

Whereas our previous analyses have focused on the trajectory of univariate, trial-wise anterior MTL and neocortical activity across recurring exposure to emotionally negative vs neutral items, previous research suggested that successful encoding of episodic memory (for neutral events) may be associated with a consistent reactivation of neural representations across repeated encounters (Xue et al., 2010, 2013; Feng et al., 2019). Whether the reinstatement of representational patterns across repeated encounters is altered for emotional information, and hence associated with a subsequent emotional enhancement of memory, remains unknown. Thus, in a next step, we applied a multivariate item-wise similarity analysis by correlating activation patterns of items in a specific presentation run with the activation patterns of the same item during a subsequent presentation run. While the MTL has been implicated in the item-level reinstatement of encoding-related activity during retrieval of neutral material (Tompary et al., 2016; Pacheco Estefan et al., 2019; Zou et al., 2023), previous literature (Strange et al., 1999; Ranganath and Rainer, 2003; Cowan et al., 2021) and the results of our univariate analyses suggest a transient role of the anterior MTL in emotional memory formation. Thus, we did not expect emotional enhancement of subsequent memory to be associated with stable activation patterns across repeated exposures in the anterior MTL. Accordingly, amygdala and anterior hippocampal pattern stability across runs was neither linked to emotional enhancement of memory (memory × emotion, all |*β*| < 0.005; all *p*_corr_ > 0.561) nor to overall memory (main effect memory, all |*β*| < 0.002; all *p*_corr_ > 0.851). Similarly, no such observation was observed in the mid or posterior hippocampus (main effect memory, |*β*| < 0.003; all *p*_corr_ > 0.565; memory × emotion, all |*β*| < 0.004; all *p*_corr_ > 0.673).

**Figure 4.**
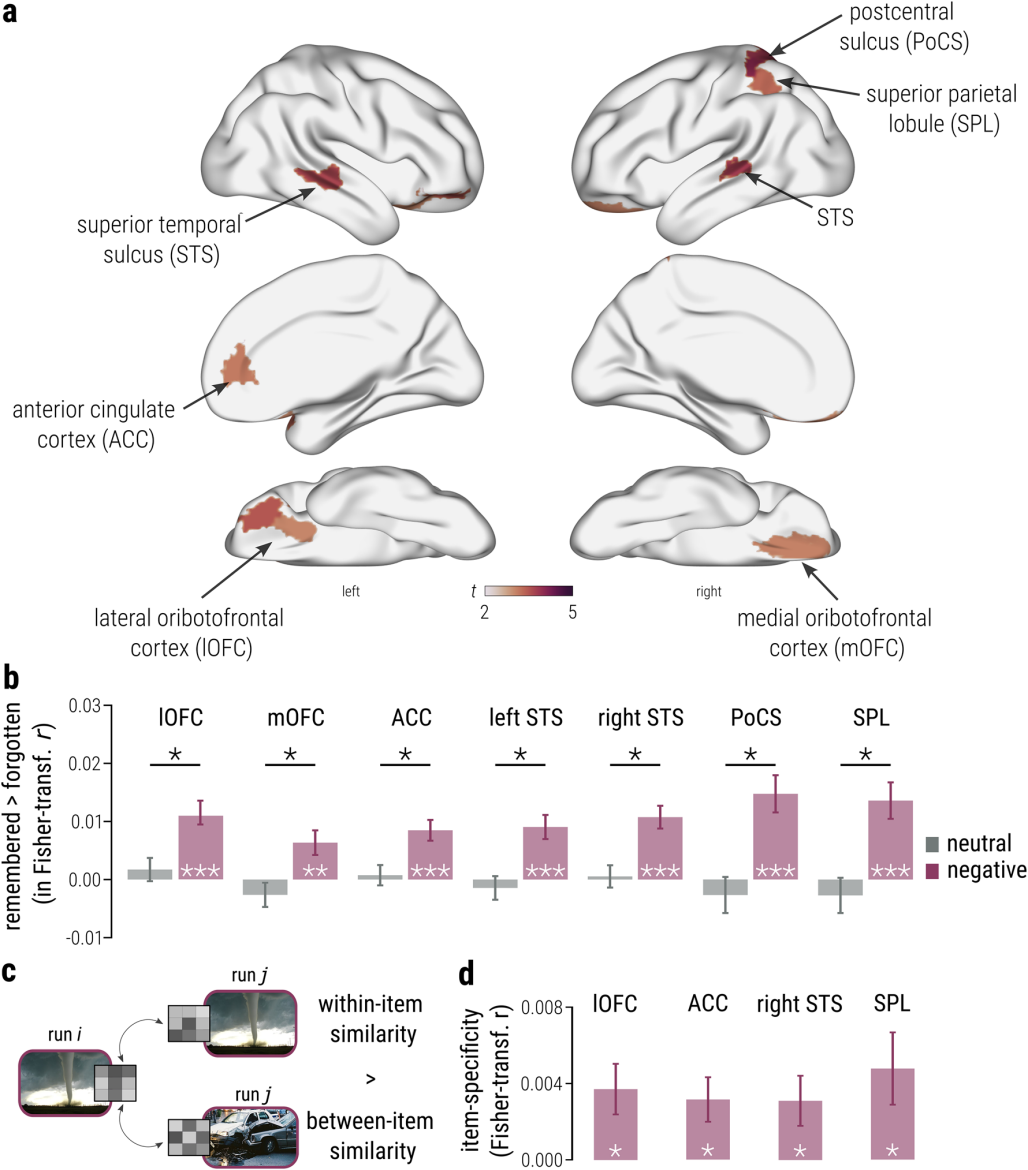
Pattern stability across repeated exposures associated with subsequent emotional memory enhancement. ***a***, Surface maps depicting the *t* statistic for the fixed effect estimate of the memory × emotion interaction in LMMs on within-item pattern similarity in each ROI, showing only regions that were significant after FDR correction. All voxels within an ROI were assigned the same value for visualization purposes. ***b***, Subsequent emotional (vs neutral) memory was associated with consistent item-wise activation patterns over repeated exposures in prefrontal (left lOFC, *p*_corr_ = 0.019; right mOFC, *p*_corr_ = 0.048; left ACC, *p*_corr_ = 0.043) and posterior neocortical areas (left STS, *p*_corr_ = 0.043; right STS, *p*_corr_ = 0.010; PoCS, *p*_corr_ = 0.010; right SPL, *p*_corr_ = 0.010; memory × emotion, LMMs). These findings highlight stable activation patterns over repeated exposures in neocortical regions associated with attention and cognitive control processes that contribute to emotional enhancement of immediate subsequent memory. Bars represent EMMs for the contrast “remembered > forgotten” per level of the predictor emotion ± SE. Black stars depict a significant memory × emotion interaction. White stars depict significant paired contrasts for forgotten versus remembered within each level of emotion representing conventional (dependent) post hoc tests of the two-way interactions shown in panel ***a***. ***c***, We further contrasted the above reported similarity between activation patterns during presentations of the same item (within-item similarity) to the similarity between activation patterns during presentation of each item and activation patterns during subsequent runs for all other items (between-item similarity) of the same subsequent memory and emotion. All included images are licensed under Creative Commons BY-SA license: the image of a tornado is courtesy of Justin Hobson (https://bit.ly/3SbJTkK; changed); the image of a car crash is courtesy of Dino Kužnik (https://bit.ly/3SodgAx; changed). ***d***, Directed post hoc contrasts of within- and between-item similarity for remembered emotionally negative items revealed significantly higher within-item similarity in the left lOFC (*p*_corr_ = 0.030), ACC (*p*_corr_ = 0.030), right STS (*p*_corr_ = 0.036), and right SPL (*p*_corr_ = 0.030) suggesting that these regions support emotional memory formation through item-specific pattern reinstatement across repetitions. Bars represent beta estimates for the contrast “within-item similarity > between-item similarity” ± SE. White stars represent significance of these contrasts. All *n* = 103. Reported *p* values are two-tailed and FDR-corrected for multiple comparisons, accounting for the number of regions of interest in each analysis (*p*_corr_). ***p < 0.001; **p < 0.010; *p < 0.010.

In contrast to MTL pattern similarity, neocortical pattern similarity across repeated encoding runs was significantly associated with subsequent memory for emotionally negative images. Specifically, recall of emotional compared with neutral images was associated with significantly higher pattern stability in the medial prefrontal cortex, including the left lateral OFC (peak, memory × emotion, *β* = 0.010; CI[0.004, 0.015]; *t*_(5114.366)_ = 3.492; *p*_corr_ = 0.019), the right medial OFC (memory × emotion, *β* = 0.009; CI[0.003, 0.015]; *t*_(6135.003)_ = 3.068; *p*_corr_ = 0.048), and the perigenual ACC (memory × emotion, *β* = 0.008; CI[0.003, 0.013]; *t*_(4803.245)_ = 3.139; *p*_corr_ = 0.043; Fig. 4*a*, middle and bottom panels; Fig. 4*b*). Notably, both the ACC and OFC have been previously implicated in emotion control processes (Etkin et al., 2011; Sakata et al., 2019) which may promote the successful encoding of emotionally arousing events. Post hoc analyses confirmed that, in both of these regions, subsequent emotional memory was associated with consistent pattern representations over repeated encounters (left OFC, EMM = 0.012; CI[0.008, 0.016]; *t*_(4414.477)_ = 5.624; *p* < 0.001; right OFC, EMM = 0.006; CI[0.002, 0.011]; *t*_(6147.45)_ = 2.997; *p* = 0.003; ACC, EMM = 0.008; CI[0.005, 0.012]; *t*_(3994.984)_ = 4.737; *p* < 0.001; Fig. 4*b*). For neutral items, there was no significant involvement of pattern stability in these prefrontal areas in subsequent memory (all |EMM| < 0.003; all *p* > 0.206; paired contrasts; Fig. 4*b*).

Moreover, subsequent memory was associated with a significantly higher pattern stability for emotionally negative compared with neutral images in the posterior section of left (memory × emotion, *β* = 0.010; CI[0.005, 0.016]; *t*_(4851.661)_ = 3.677; *p*_corr_ = 0.012) and right STS (memory × emotion, *β* = 0.010; CI[0.005, 0.016]; *t*_(4664.417)_ = 3.794; *p*_corr_ = 0.010; Fig. 4*a*, top panel; Fig. 4*b*). Although this appears to mirror our univariate findings of more stable activations for subsequently remembered emotionally negative compared with neutral events in posterior temporal cortices, it is crucial to note that regions exhibiting a significant effect in our univariate analyses did not overlap with regions showing a significant neocortical pattern stability, ruling out that our multivariate findings might be driven by univariate activation differences. Subsequent memory for emotionally negative (vs neutral) items was further associated with significantly more stable pattern representations in posterior parietal regions, such as the right postcentral gyrus (memory × emotion, *β* = 0.017; CI[0.009, 0.026]; *t*_(5822.193)_ = 4.002; *p*_corr_ = 0.010) and right SPL (peak, memory × emotion, *β* = 0.016; CI[0.008, 0.025]; *t*_(5930.284)_ = 3.836; *p*_corr_ = 0.010; Fig. 4*a*, top-right panel; Fig. 4*b*). Notably, the SPL, as part of the dorsal attentional network (Corbetta and Shulman, 2002; Corbetta et al., 2008; Spreng et al., 2013), is expected to foster perceptual attentional processes that support memory formation (Sestieri et al., 2017). The postcentral sulcus, interconnected with the frontoparietal control network (Vincent et al., 2008), on the other hand, is expected to support memory encoding by consistent evaluation of the familiarity of the presented material (Sestieri et al., 2017). Our results did not indicate any statistically significant association between more dissimilar, i.e., more variable, encoding patterns and subsequent memory for emotionally negative nor neutral images.

We further explored whether the observed neocortical pattern similarities reflect item-specific pattern reinstatement or more general activation patterns associated with successful encoding of emotional material. For this, we contrasted the above reported similarity between activation patterns during presentations of the same item (within-item similarity) to the similarity between activation patterns during presentation of each item and activation patterns during subsequent runs for all other items (between-item similarity) of the same subsequent memory (forgotten vs remembered) and emotion (neutral vs negative) category. Analyzing item-wise encoding pattern stability with the factors emotion (neutral vs negative), subsequent memory (forgotten vs remembered), and item specificity (between-item vs within-item) indicated a significant memory × emotion × item specificity interaction in the ACC (*β* = 0.007; CI[0.002, 0.012]; *t*_(12188.81)_ = 2.615; *p*_corr_ = 0.018) and OFC (left, *β* = 0.015; CI[0.006, 0.024]; *t*_(12189.31)_ = 3.157; *p*_corr_ = 0.016; right, *β* = 0.008; CI[0.002, 0.014]; *t*_(12246.88)_ = 2.640; *p*_corr_ = 0.018). Similarly, the right SPL (*β* = 0.010; CI[0.001, 0.018]; *t*_(12188.52)_ = 2.148; *p*_corr_ = 0.040), postcentral gyrus (*β* = 0.012; CI[0.003, 0.02]; *t*_(12188.64)_ = 2.548; *p*_corr_ = 0.018), and right posterior STS (*β* = 0.008; CI[0.002, 0.013]; *t*_(12188.29)_ = 2.758; *p*_corr_ = 0.018) showed a significantly higher within-item compared with between-item similarity associated with subsequent emotional memory enhancement (memory × emotion × item specificity interaction). The left STS did not show a significant difference between within- and between-item pattern stability for subsequently remembered emotionally negative items (memory × emotion × item specificity, *p*_corr_ = 0.139), indicating this region may rather support general processes associated with subsequent emotional memory, as indicated in the main pattern stability analysis, rather than item-specific pattern reinstatement.

Importantly, a direct comparison of within- and between-item similarity by means of follow-up LMMs focusing on remembered emotionally negative items (Fig. 4*c*) revealed significantly higher within-item similarity in the left OFC (*β* = 0.004; *t*_(3619.129)_ = 2.803; *p*_corr_ = 0.030), left ACC (*β* = 0.003; *t*_(3621.736)_ = 2.699; *p*_corr_ = 0.030), right STS (*β* = 0.003; *t*_(3617.737)_ = 2.365; *p*_corr_ = 0.036), and right SPL (*β* = 0.005; *t*_(3617.953)_ = 2.518; *p*_corr_ = 0.030; Fig. 4*d*), suggesting that these regions support emotional memory formation through item-specific pattern reinstatement across repetitions. The right medial OFC (*p*_corr_ = 0.498), left STS (*p*_corr_ = 0.113), and right postcentral sulcus (*p*_corr_ = 0.093) did not exhibit a significant within- versus between-item difference, indicating that these areas may instead support more general processes related to subsequent emotional memory formation.

Consistent with this interpretation, analyzing between-item similarity by means of follow-up LMMs with the factors emotion (neutral vs negative) and subsequent memory (forgotten vs remembered) revealed a significant memory × emotion interaction in the postcentral gyrus (*β* = 0.008; *t*_(5710.795)_ = 4.370; *p*_corr_ < 0.001) as well as in the right SPL (*β* = 0.009; *t*_(5775.765)_ = 5.295; *p*_corr_ < 0.001) and with a similar trend for the right STS (*β* = 0.003; *t*_(5208.704)_ = 2.239; *p*_corr_ = 0.057). These results suggest that posterior regions like the SPL and STS, while showing item-specific pattern reinstatement (memory × emotion × item specificity interaction), also contribute to category-level reinstatement, supporting subsequent emotional memory through more general activation patterns. This analysis did not indicate a memory × emotion interaction in the ACC (*p*_corr_ = 0.276) or bilateral OFC (left, *p*_corr_ = 0.992; right, *p*_corr_ = 0.496), indicating that the previously observed neocortical pattern stability in these areas may be specifically related to the reinstatement of the representation of each item across repetitions.

Thus, results of our multivariate analyses suggest that subsequent memory for recurring negative events is associated with stable pattern representations over repetitions in prefrontal and posterior parietal areas, which have been previously linked to attentional and cognitive control processes. These findings align with previous work suggesting a crucial role of cognitive factors, such as heightened attention to emotionally negative material when encoding emotional material (Talmi, 2013), and further indicate the role of item-specific pattern reinstatement in prefrontal cortices in the emotional enhancement of immediate memory.

### Amygdala activity at first exposure modulates emotional memory enhancement via neocortical pattern stability over repetitions

As a final step, we analyzed whether anterior MTL engagement at initial exposure, i.e., Presentation Run 1, influences immediate emotional memory enhancement via stable neocortical pattern activations over repeated encounters. To this end, we employed a multilevel-moderated mediation analysis with the predictor emotion (neutral vs negative), the outcome variable subsequent memory (forgotten vs remembered), the mediator superior parietal pattern stability, and the moderator anterior MTL (left amygdala or right anterior hippocampus) activity at initial encounter. This approach involved (1) an LMM estimating the mediator as a function of the predictor, the moderator, and their interaction; (2) a generalized LMM predicting the outcome by means of the predictor and mediator, both interacting with the moderator; (3) evaluating the moderated mediation by assessing conditional indirect effects at different levels of the moderator (Tingley et al., 2014; Hayes, 2015); and (4) inference statistical testing of the linear change of conditional indirect effects as a linear function of the mediator—the index of moderated mediation (Hayes, 2015)—via bias-corrected bootstrap confidence intervals (as recommended by Hayes, 2015).

**Figure 5.**
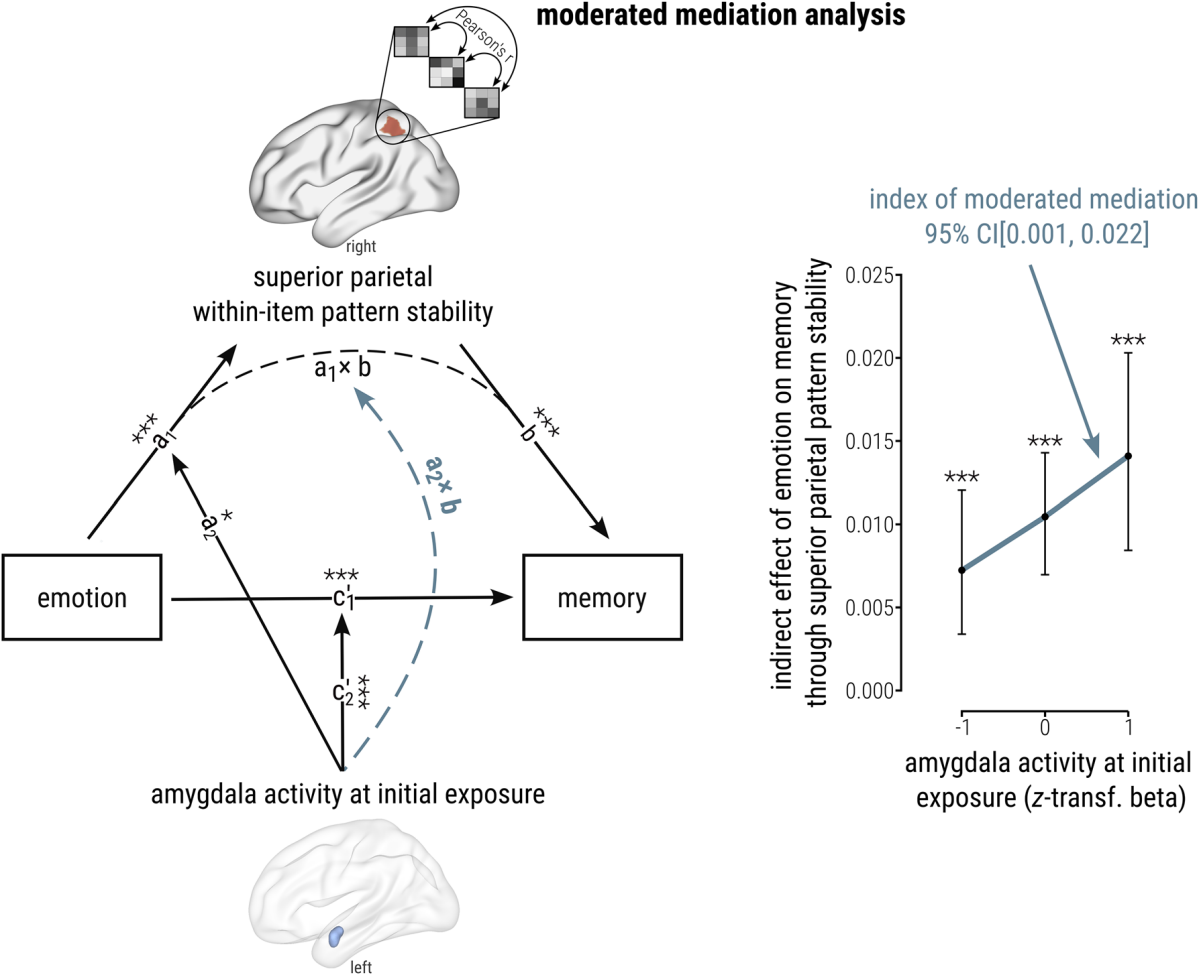
Amygdala activity at first exposure boosts subsequent emotional memory via neocortical pattern stability. Left panel, Multilevel-moderated mediation analyses revealed a significant mediation of emotional memory enhancement through superior parietal pattern stability (indirect effect, a_1 _× b, *p*_corr_ < 0.001; direct effect, path $c_{1}^{\prime }$, *p*_corr_ < 0.001). Right panel, The indirect effect of emotion on subsequent memory through stable neocortical encoding patterns was significantly facilitated by amygdala activity during initial stimulus exposure, as indicated by the index of moderated mediation (a_2 _× b). These findings underscore the pivotal role of initial amygdala engagement and its interaction with the SPL in facilitating memory for recurring emotional events. Inference statistical testing of the index of moderated mediation based on bias-corrected, bootstrapped confidence interval (as recommended by Hayes, 2015). Points represent conditional indirect effects at low (−1 SD), mean, and high (+1 SD) levels of the moderator ± SE. The regression line illustrates the index of moderated mediation, i.e., the linear change in the indirect effect as a function of the moderator. All *n* = 103. Reported *p* values are two-tailed and FDR-corrected for multiple comparisons, accounting for the number of regions of interest in this analysis (*p*_corr_). ***p < 0.001; *p < 0.050.

This analysis revealed a direct link between amygdala activity at initial exposure and consistent pattern activations over repeated encoding facilitating emotional enhancement of immediate free recall. When controlling for the influence of the moderator (initial amygdala activity), superior parietal pattern stability significantly mediated emotional enhancement of subsequent memory (indirect effect; Fig. 5, $\;a_{1}\times b$; *β* = 0.010; CI[0.007, 0.014]; *p*_corr_ < 0.001). When controlling for this indirect effect through superior parietal pattern stability (and the moderator), the direct effect of emotion on immediate subsequent memory remained significant (Fig. 5, $c_{1}^{\prime }$; *β* = 0.156; CI[0.133, 0.180]; *p*_corr_ < 0.001), suggesting a partial mediation by consistent encoding patterns in the SPL. Critically, the extent of amygdala activation at initial encoding significantly moderated this relationship (index of moderated mediation, $a_{2}\times b$; *β* = 0.013; CI[0.001, 0.022]; Fig. 5, right panel), with higher amygdala activity (*z*-transformed beta = 1) enhancing the indirect effect of emotion on memory via superior parietal pattern stability (compared with lower levels of initial amygdala activity; indirect effect, *β* = 0.014; CI[0.008, 0.020]; *p*_corr_ < 0.001; direct effect, *β* = 0.186; CI[0.152, 0.219]; *p*_corr_ < 0.001). Conversely, if amygdala activity during initial exposure was relatively low (*z*-transformed beta = −1), emotional memory enhancement through stable posterior parietal patterns was reduced (indirect effect, *β* = 0.007) but still significant (CI[0.003, 0.012]; *p*_corr_ < 0.001; direct effect, *β* = 0.125; CI[0.092, 0.159]; *p*_corr_ < 0.001). No other region but the SPL indicated a significant moderated mediation by initial anterior MTL activity. Notably, repeating this analysis with between-item SPL pattern similarity did not reveal a significant moderated mediation by amygdala activity at initial exposure (index of moderated mediation, CI[−0.005, 0.024]), suggesting that the observed effect is specific to item-specific SPL pattern reinstatement rather than category-general reinstatement. These results indicate that amygdala activation during the initial encounter with an emotional stimulus may enhance subsequent immediate memory for that stimulus through persistent, item-specific superior parietal pattern reinstatement across repeated exposures.

## Discussion

This study aimed to elucidate the neural mechanisms underlying memory formation for recurrently encountered emotional events. Our findings reveal that successful emotional memory formation is linked to persistent activation patterns in neocortical attention and cognitive control regions, coupled with transient activation in anterior MTL and temporofrontal regions upon first encountering an emotional event. Critically, the extent of amygdala activation during this first exposure facilitated emotional memory enhancement via neocortical pattern stability.

The observed elevation in amygdala and anterior hippocampal activity during the first exposure to an emotionally negative item corroborates previous findings highlighting the pivotal role of these regions in detecting novel, salient stimuli (Ranganath and Rainer, 2003; Cowan et al., 2021) and emotional memory encoding (Murty et al., 2011; Dahlgren et al., 2020). Rapid detection of emotionally salient events is crucial for survival in potentially threatening situations and has been predominantly linked to the amygdala (LeDoux and Phelps, 2008). Notably, our findings demonstrate a transient involvement of the anterior MTL emotional memory formation, with an activation decrease over repeated exposures. This change in activity dovetails with the adaptive response of the anterior MTL during emotional learning (Büchel and Dolan, 2000; Yin et al., 2018), which may be at least partly due to a habituation to emotional stimuli. Moreover, this trajectory of a decrease in anterior MTL (amygdala and anterior hippocampus) activity across repetitions was mirrored by anterior temporal areas and the right IFG, which have been linked to attention allocation based on stimulus salience (Corbetta et al., 2008), emotional memory enhancement (Dolcos et al., 2004; Ritchey et al., 2011; Weintraub-Brevda and Chua, 2018), novelty detection (Ranganath and Rainer, 2003), and semantic memory (Patterson et al., 2007; Binder and Desai, 2011; Ralph et al., 2017). These areas may promote efficient processing of novel, emotionally salient information by anchoring it to existing knowledge frameworks (Prince et al., 2007; Irish and Piguet, 2013; Sommer, 2016). In sharp contrast to the change in activity in anterior temporofrontal regions, our results indicate that subsequent memory for recurring neutral items is marked by increasing involvement of posterior temporal and parietal cortices associated with semantic processes (Patterson et al., 2007; Binder and Desai, 2011; Liebenthal et al., 2014; Ralph et al., 2017). The increased engagement of angular gyrus over repeated exposure aligns with previous findings associating this region with cortical memory formation in the course of repeated spatial learning (Brodt et al., 2016). While the angular gyrus has been repeatedly associated with memory retrieval (Sestieri et al., 2017), it is likely to reflect more generalized, schema-based memories (Binder et al., 2009; Wagner et al., 2015; van der Linden et al., 2017). Thus, in the absence of emotional salience, memory formation may rely on repeated encounters, potentially leading to more generalized memory representations (Nadel and Moscovitch, 1997; van der Linden et al., 2017; Hebscher et al., 2019). Thus, our data suggest that this repetition-based memory formation in posterior parietal areas such as the angular gyrus may be slowed down for emotionally negative events, potentially in order to keep more specific representations of these events. Importantly, however, this idea remains speculative, as we did not test for generalized or schema-like representations in the present study. Moreover, it has to be noted that, although participants were told to memorize the images during the consecutive presentation runs, it is likely that Runs 2 and 3 reflect both stimulus encoding and retrieval-related activity. Thus, especially the observed increase in memory-related activity for neutral items across runs might potentially reflect successful retrieval which may benefit later memory.

While our trial-wise univariate analyses focused on activation changes over repeated encounters, our multivariate analyses allowed us to probe the stability (vs variability) of activation patterns across repetitions. Previous findings (Xue et al., 2010, 2013; Feng et al., 2019) indicated that successful memory formation for neutral events is associated with the consistent reinstatement of similar representation patterns over repetitions rather than an increased dissimilarity over repeated encounters as suggested by encoding-variability accounts (Estes, 1955; Bower, 1972; Johnston, 1976). Here, we show that this pattern stability across repetitions is even enhanced for emotional compared with neutral events, particularly in prefrontal regions such as the ACC and OFC. These persistent prefrontal activation patterns may reflect the involvement of these regions in facilitating attention to emotional stimuli (Seeley et al., 2007; Pourtois et al., 2013), which, in turn, may enhance subsequent memory for emotional events (Kensinger, 2009; Mather and Sutherland, 2011; Pourtois et al., 2013). Accordingly, subsequent memory for emotional (compared with neutral) images was furthermore associated with more consistent activation patterns in the SPL, likely reflecting sustained perceptual attention to emotionally negative events (Corbetta and Shulman, 2002; Corbetta et al., 2008; Spreng et al., 2013). Interestingly, exploring the item specificity of these effects in the ACC and OFC indicated that the increased pattern stability associated with emotional enhancement of subsequent memory was specific to pattern correlations within items and not observed when correlating pattern activations across items. This suggests that these prefrontal regions may support emotional memory formation through item-specific pattern reinstatement across repeated encounters. However, in other regions, such as the SPL or STS, between-item pattern stability was linked to subsequent emotional memory. This suggests that these regions are involved in more general processes, such as attention or emotional states, which may support emotional memory formation. These findings, indicating an increased pattern stability for subsequently recalled emotional material, are furthermore consistent to previous findings associating emotional arousal with enhanced similarity between encoding and retrieval patterns (Ritchey et al., 2013) and enhanced reinstatement of activity patterns during initial learning of face–object associations when subsequently presented with overlapping faces coupled with neutral vs aversive tones (Zhu et al., 2022).

Intriguingly, the results of our multilevel-moderated mediation analysis indicate that the amygdala’s transient response at initial event exposure may boost subsequent memory for emotionally salient information via persistent superior parietal (re)activation patterns across recurring exposures. This finding is consistent with previous reports suggesting that the amygdala guides perceptual attention to emotionally salient information through interactions with frontoparietal attention cortices (Liberzon et al., 2003; Lim et al., 2009; Mather and Sutherland, 2011; Pourtois et al., 2013). Thus, these results support previous accounts suggesting that emotional enhancement of immediate memory may be mediated by cognitive factors such as increased perceptual attention to emotionally salient information (Pourtois et al., 2013; Talmi, 2013) while highlighting the modulation of this process by amygdala activation at initial exposure. This dynamic may be highly adaptive as it, on the one hand, ensures sustained attention to and, consequently, subsequent remembering of emotionally salient events (Pourtois et al., 2013) while allowing the amygdala to reset and respond to potential novel, emotionally salient information. Notably, while previous accounts implicated increased involvement in visual cortices during emotional memory encoding (Mather and Sutherland, 2011; Pourtois et al., 2013), neither our univariate nor our multivariate analyses indicated beneficial activation (patterns) in such regions when presented with subsequently remembered emotional (compared with neutral) images across repetitions.

While the current study focused on the neural underpinnings of successful encoding of repeatedly encountered emotional events by presenting stimuli in close temporal proximity, real-life scenarios often involve longer delays between encounters. Such increased delays may be associated with neural reorganization of memories from previous encounters (Winocur et al., 2010; Squire et al., 2015), with previous research suggesting that time-dependent memory transformation may be particularly facilitated for emotionally arousing stimuli (Dandolo and Schwabe, 2018; Krenz et al., 2023). Additionally, everyday experiences typically involve variations in spatial context and other relevant factors rather than repeated exposure to identical stimuli. Consequently, future studies should explore the effects of varying delays and contextual factors on the neural mechanism underlying memory formation for recurrent emotional events identified here. Moreover, future studies should intermix repeated items with novel items across runs, to allow investigating whether the anterior MTL increase observed in Run 1 would similarly be elicited by novel items introduced in subsequent runs.

To conclude, our findings shed light on the dynamic interplay between transient amygdala activation and persistent neocortical activation patterns in the formation of memories for recurring emotional events. Moreover, our data indicate distinct trajectories of neocortical activity over repetitions of emotional and neutral events with a diminishing engagement in anterior temporofrontal regions for emotionally salient stimuli and a progressive increase in posterior temporal and parietal activation for neutral events. Beyond their relevance for our understanding of the evolution of emotional memory across repeated encounters, these findings might have implications for the development of novel interventions for disorders such as complex post-traumatic stress disorder that are characterized by recurring traumatic events and the debilitating memories thereof.
